# Emerging Pathogens Causing Acute Hepatitis

**DOI:** 10.3390/microorganisms11122952

**Published:** 2023-12-10

**Authors:** Ibrahim M. Sayed, Ahmed El-Shamy, Sayed F. Abdelwahab

**Affiliations:** 1 Department of Biomedical & Nutritional Sciences, University of Massachusetts Lowell, Lowell, MA 01854, USA; 2 Master of Pharmaceutical Sciences Program, College of Graduate Studies, California Northstate University, Elk Grove, CA 95757, USA; ahmed.elshamy@cnsu.edu; 3 Department of Pharmaceutics and Industrial Pharmacy, College of Pharmacy, Taif University, Taif 21944, Saudi Arabia; s.fekry@tu.edu.sa

Acute hepatitis is defined as an inflammation or injury in the hepatocytes that continues for a short period of time (less than 6 months) [1]. There are several causes for acute hepatitis which can be divided into microbial and non-microbial factors. Non-microbial causes of acute hepatitis include alcohol-induced hepatitis [2], drugs such as acetaminophen and non-steroidal anti-inflammatory drugs [3], toxins such as mushrooms [4], fatty liver diseases [5], autoimmune hepatitis [6], metabolic or hereditary causes such as Wilson’s disease [7], and pregnancy-associated hepatitis [8], etc. Microbes also cause acute hepatitis. Most of the reported hepatitis cases are caused by hepatitis viruses (A-E viruses) [9]. However, non-hepatotropic viruses such as human cytomegalovirus (CMV), human adenovirus, human herpes virus 6, varicella-zoster virus, and Epstein–Barr virus (EBV) were also reported as causes of acute hepatitis [10]. Bacteria and fungi are also associated with acute-on-chronic liver failure, and these infections lead to poor prognosis and high death rates [11].

In 2019, viral hepatitis caused 1.57 million deaths globally [12]. Hepatitis viruses (HAV, HBV, HCV, HDV, and HEV) are the most documented causes of acute hepatitis, which could progress to acute liver failure [9]. HBV and HCV also cause chronic infections that can lead to liver cirrhosis and cancer [13,14]. HEV also causes chronic infections, especially with genotypes 3 and 4, which could be linked to extrahepatic disorders such as neurological abnormalities, kidney dysfunctions, and blood cell diseases [15]. The transmission routes of these viruses are either through the parenteral route (HBV, HCV, HDV, and HEV) or the oral route (HAV and HEV) [16]. HAV and HEV infections cause epidemics and outbreaks, especially in developing countries, where the lack of hygienic practices, education and awareness, and hepatitis A vaccine programs could increase the prevalence rate [17]. In this issue, Kayesh and colleagues reported the epidemiology and risk factors of viral hepatitis in Bangladesh [16]. They reported that HAV and HEV are the main causes of acute viral hepatitis in Bangladesh, and the implantation of an effective vaccine and good hygiene plans could significantly reduce the infection rate [16]. A nationwide surveillance was performed on 998 patients enrolled in 10 different hospitals in Bangladesh, revealing that HAV accounted for 19% of infections, especially in children, and HEV caused 10% of infections, especially among adults [18]. In addition, Kayesh et al. reported that the actual prevalence of HAV and/or HEV in Bangladesh is still underestimated [16]. Besides the application of preventive strategies, routine diagnosis of these two viruses in Bangladesh could help in the documentation of the actual estimates of infection with these two viruses and/or possible coinfection cases.

Viral hepatitis can affect pregnancy, and the vertical transmission of hepatitis viruses, especially HBV, HCV, and HEV, was reported [19]. The course of HBV and HCV during pregnancy is either acute or chronic, and the preterm delivery and complications are mainly seen with HBV infection [19,20,21]. The course of HAV during pregnancy is benign and self-limiting, and the complications to the fetus are rare [22]. Most complications during pregnancy are due to HEV infection which causes acute or fulminant hepatitis in pregnant women, and fetal death, stillbirth, and/or preterm delivery were reported [23]. HEV genotypes affect the outcomes of infection. HEV genotype 1 is virulent and its infection is severe, especially if the infection occurs in the third trimester [24], while HEV genotype 3 is not severe during pregnancy [25,26]. However, the pathogenesis of HEV infection during pregnancy is not fully understood. In this issue, Yadav and Kenney discussed the animal models used to study the congenital transmission of HEV to understand the pathogenesis of HEV during pregnancy [27]. The authors first described the immunological and hormonal changes during pregnancy [27]. There is no ideal animal model that can study HEV pathogenesis during pregnancy from all aspects, though non-human primates could be suitable for studying HEV genotypes 1–4 [27]. Rabbits and pigs could be suitable models for studying the effect of chronic HEV infection on pregnancy [27]. Although animal models are considered appropriate candidates, these models could not completely recapitulate the pregnancy in humans. Recent studies on HEV pathogenesis showed that primary cells isolated from the maternal fetus interface, decidualized and non-decidualized stromal cells support HEV replication [28,29]. However, the primary cells have limited in vitro propagation ability. It is probable that 3D stem-cell-based organoids isolated from pregnant women can propagate higher passages and could physiologically mimic the pregnancy cycle in vivo. 

Non-hepatotropic viruses also cause acute hepatitis. In 2022, the World Health Organization alerted us of more than one thousand cases and twenty deaths from acute hepatitis in children that are not caused by hepatitis viruses (A–E) [30]. Adenoviruses and severe acute respiratory syndrome corona virus-2 (SARS-CoV-2) were the common cause of acute hepatitis of unknown etiology in children [31,32]. Adenoviruses not only infect children, but they also infect adults, causing acute hepatitis, chronic hepatitis, and/or fulminant hepatitis, either alone or in coinfection with HEV [33]. In this issue, Săndulescu and colleagues performed a retrospective study over the past five years on 1416 children with confirmed adenovirus infection in Romania [34]. The authors reported that the digestive symptoms, especially diarrhea, were common symptoms and the aspartate transaminase enzyme was more elevated than alanine transaminase in those children [34]. Importantly, the authors showed that 31% of the cases (n = 446) were coinfected cases [34]. Digestive microbes were reported in 86% of coinfected cases (n = 402), mainly rotavirus, *Campylobacter* spp., and *Salmonella* spp. [34]. On the other hand, non-digestive microbes such as respiratory viruses including influenza virus, SARS-CoV-2, respiratory syncytial virus, and rhinovirus, and other viruses such as CMV, EBV, and measles, represented the remaining coinfection cases [34]. Interestingly, several cases of bacterial infections were documented with acute hepatitis such as *Campylobacter* spp., *Salmonella* spp., *Shigella* spp., and *Yersinia enterocolitica* [34]. Bacterial infections are underestimated causes of acute hepatitis since most of the bacteria are not routinely enrolled in diagnosis in the cases of acute hepatitis. In this regard, El-Mokhtar and colleagues tested hepatitis cases of unknown etiology (n = 67) for *Coxiella burnetii*, the causative agent of Q-fever. The authors found four positive cases of Q-fever; one case was coinfected with HEV and the remaining three cases were only infected with *Coxiella burnetii* [35]. The authors concluded that enrollment of Q-fever diagnosis in the case of acute hepatitis could be beneficial in the early identification of the disease and for taking the appropriate measures [35].

Coinfection between hepatotropic viruses is documented in many developing countries [36]. HAV and HEV have common features including mode of transmission, genomic organizations, and pathogenesis [36]. Therefore, co-infection of these two viruses is common. However, the outcomes of this co-infection are not known. In this issue, EL-Mokhtar and colleagues reported that 36.8% of HAV-infected patients were HAV-HEV-coinfected ones [37]. The liver function profile was comparable in the HAV-HEV coinfected patients and HAV mono-infected ones. However, the risk of acute liver failure was higher among the coinfected patients [37]. 

In conclusion, several pathogens cause acute hepatitis including viral and bacterial hepatitis. Not all the microbes are routinely diagnosed in acute hepatitis cases. Therefore, several types of pathogen-mediated acute hepatitis are still underestimated. Next-generation sequencing approaches could help in the identification of emerging pathogens causing acute hepatitis. Microbe-mediated acute hepatitis could progress to acute liver failure or other liver diseases. The early identification of the causative agent will help clinicians in prescribing effective therapies for their patients.
